# Supplementary material for: “What do I think about implementing lung cancer screening? It all depends on how.” Acceptability and feasibility of lung cancer screening in Australia: The view of key stakeholders about health system factors
Source: PLoS One. 2023 Apr 5;18(4):e0283939. doi: 10.1371/journal.pone.0283939 (PMC10075440; doi:10.1371/journal.pone.0283939)
Supplement: S1 File — (DOCX) [file pone.0283939.s001.docx]

**Supplementary file1: Completed COREQ Criteria Checklist for manuscripts.**

| **Domain 1: Research team and reflexivity**  ***Personal Characteristics*** | **Covered in article?** | **Page** |
| --- | --- | --- |
| 1. Interviewer/facilitator Which author/s conducted the interview or focus group? | Yes (Y) | 8 |
| 2. Credentials What were the researcher’s credentials? E.g. PhD, MD | Y | 8 |
| 3. Occupation What was their occupation at the time of the study? | Y | 8 |
| 4. Gender Was the researcher male or female? | Y | 8 |
| 5. Experience and training: What experience or training did the researcher have? | Y | 8 |
| ***Relationship with participants*** |  |  |
| 6. Relationship established: Was a relationship established prior to study commencement? | - Y | 7 |
| 7. Participant knowledge of the interviewer? What did the participants know about the researcher? e.g. personal goals, reasons for doing the research | - Y | 7 |
| 8. Interviewer characteristics What characteristics were reported about the interviewer/facilitator? e.g. Bias, assumptions, reasons and interests in the research topic. | - Y | 7 |
| **Domain 2: study design**  ***Theoretical framework*** |  |  |
| 9. Methodological orientation and Theory  What methodological orientation was stated to underpin the study? e.g. grounded theory, discourse analysis, ethnography, phenomenology, content analysis. | Y | 8 |
| ***Participant selection*** |  |  |
| 10. Sampling How were participants selected? e.g. purposive, convenience, consecutive, snowball | - Y | 6 |
| 11. Method of approach How were participants approached? e.g. face-to-face, telephone, mail, email | - Y | 6 |
| 12. Sample size How many participants were in the study? | - Y | 9 |
| 13. Non-participation How many people refused to participate or dropped out? Reasons? | No (N) | Not applicable (NA) |
| ***Setting*** |  |  |
| 14. Setting of data collection  Where was the data collected? e.g. home, clinic, workplace | - Y | 8 |
| 15. Presence of non-participants Was anyone else present besides the participants and researchers? | - N | NA |
| 16. Description of sample What are the important characteristics of the sample? e.g. demographic data, date | - Y | 9 |
| ***Data collection*** |  |  |
| 17. Interview guide Were questions, prompts, guides provided by the authors? Was it pilot tested? | Y | 7 |
| 18. Repeat interviews Were repeat interviews carried out? If yes, how many? | N | NA |
| 19. Audio/visual recording Did the research use audio or visual recording to collect the data? | - Y | 8 |
| 20. Field notes: Were field notes made during and/or after the interview or focus group? | N | NA |
| 21. Duration What was the duration of the interviews or focus group? | - Y | 8 |
| 22. Data saturation: Was data saturation discussed? | - N |  |
| 23. Transcripts returned Were transcripts returned to participants for comment and/or correction? | N | NA |
| **Domain 3: analysis and findings**  *Data analysis* |  |  |
| 24. Number of data coders  How many data coders coded the data? | - Y | 8 |
| 25. Description of the coding tree  Did authors provide a description of the coding tree? | Y | 8 |
| 26. Derivation of themes  Were themes identified in advance or derived from the data? | - Y | 8 |
| 27. Software  What software, if applicable, was used to manage the data? | - Y | 8 |
| 28. Participant checking  Did participants provide feedback on the findings? | N | NA |
| ***Reporting*** |  |  |
| 29. Quotations presented Were participant quotations presented to illustrate the themes / findings? Was each quotation identified? e.g. participant number | - Y | 19-31 |
| 30. Data and findings consistent Was there consistency between the data presented and the findings? | - Y | 8 |
| 31. Clarity of major themes Were major themes clearly presented in the findings? | - Y | 19-31 |
| 32. Clarity of minor themes Is there a description of diverse cases or discussion of minor themes? | - Y | 19-31 |
