# Supplementary material for: “What do I think about implementing lung cancer screening? It all depends on how.” Acceptability and feasibility of lung cancer screening in Australia: The view of key stakeholders about health system factors
Source: PLoS One. 2023 Apr 5;18(4):e0283939. doi: 10.1371/journal.pone.0283939 (PMC10075440; doi:10.1371/journal.pone.0283939)
Supplement: S2 File — (DOCX) [file pone.0283939.s002.docx]

**S2. Supplementary file: Coding framework: Generation of topics across all factors**

**Health system factors (5 topics)**

**Complexity of health systems (9 items)**

1. Complexity of health systems (general)
2. Competition between screening programs
3. Federal and state divide
4. Harder to access radiology in regional areas
5. Healthcare administration
6. Involve all stakeholders
7. LC heterogenous
8. Public and private providers
9. Resistance to LCS

**Cost (12 items)**

1. Cost (general)
2. Are there better ways to reduce LC?
3. Balance initial investment with long term cost savings
4. Cost to set up (infrastructure)
5. Costs of outreach and funding model for centres offering LCS
6. Medicare for more than just scan
7. Opportunity costs
8. Out of pocket costs (for participants)
9. Personal and emotional cost (for participants)
10. Reduce use of expensive treatments
11. Self-referral (would) reduce costs
12. Use of existing resources to save costs

**Delivery** **of the program (22 items)**

1. Delivery of the program (general)
2. Access
3. Agreed protocol
4. American experience
5. Bridge government to clinical
6. Federal and state divide (of program delivery)
7. Gradual implementation
8. Harness lessons from other screening programs
9. Key Performance Indicators
10. Lessons from COVID
11. Limited resources
12. Link with local clinics and early assessment clinics
13. Logistics of operation
14. Need champions
15. Need infrastructure and data management
16. Population based screening framework
17. Recognise access challenges of each state
18. Referral for incidental findings
19. Start with those hard to reach
20. Support for UK model
21. Telehealth
22. Well defined pathway

**Quality assurance (18 items)**

1. Quality assurance (general)
2. Other aspects of quality assurance
3. Accreditation for reading images
4. Clinical history impact
5. Consensus statements, evidence
6. Cost (of quality assurance)
7. GP Software
8. LCS already happening?
9. Lung RADS
10. National register
11. Providing volumetrics
12. Quality frameworks
13. Quality of scanners
14. Reputation of clinics and program
15. Retraining
16. Scan interpretation guidelines, artificial intelligence (AI)
17. Sub-set of radiologists with credentials
18. Support from AI

**Workforce considerations (26 items)**

1. Workforce considerations (general)
2. Challenges in diagnostic stage
3. Clinical guidelines
4. Consultations with clinical staff
5. Early detection, different resources
6. Flow on effects of detection
7. General Practice
8. Good reimbursement
9. GPs as gatekeepers need education
10. Legal issues
11. Longer term less patients, better outcomes
12. Medical oncologists
13. Overwhelm system
14. Primary health networks
15. Providers, subcontractors
16. Psychologists
17. Radiographers
18. Radiologists
19. Radiology clinics with dedicated slots
20. Respiratory physicians
21. Rural, remote, travelling
22. Small number of biopsy clinics
23. Stagger eligible over number of years
24. Understand where workforce planning needed
25. Upscaling of current protocols
26. Work with local clinics

**Cross cutting themes (5 topics – 4 in HSF paper)**

**Access and equity (13 items)**

1. Access and equity (general)
2. Access to scanners
3. Access to treatment
4. Autonomy
5. Bring to program to the people
6. Convenience of screening
7. Digital literacy and equity
8. Ensuring those most at risk can get access
9. Inner city more awareness of risk
10. Mobile screening vans
11. Multipronged approach to outreach
12. Not exacerbate inequities
13. Radiology more expensive, harder to access regional

**Communication and outreach (9 items)**

1. Communication and outreach (general)
   1. : Build trust
2. Consumer input
3. Educate everyone
4. Marketing is key
5. Need multifaced ways to reach population
6. Outreach to all, not just those high risk
7. Participant champions
8. Simple methods
9. Understand the population

**Enthusiasm for screening (12 items)**

1. Enthusiasm for screening (general)
2. Already discussions internally - politics
3. COVID shown us what we can do from distance
4. People who have quit smoking are keen
5. Invest money elsewhere
6. Involve target population early in planning
7. Lung cancer survivors
8. Potential uptake
9. Screening fatigue
10. People who smoke – motivation
11. Support from health professionals
12. Using X-ray to screen

**Referral across the program (14 items)**

1. Referral across program (general)
2. Central database for scan access
3. Centralise the system
4. Entry into lung or imaging MDT
5. Entry points
6. GP referral
7. Incidental findings
8. Need appropriate follow up, guidelines
9. Need timely referral for investigations
10. Specialist interventions
11. Supportive follow up structure
12. Timeline, targets
13. Who communicate screening results
14. Work with health structures already in place

**Smoking cessation (35 items) (separate analysis)**

1. Smoking cessation (general)
2. Comparisons
3. Consenting to program
4. Consider stigma
5. Consumer input
6. Cost to set up
7. Downstream impact of referral out of program
8. Emotional state
9. Existing programs
10. Family pressure
11. GP time restraints
12. Impact screening
13. Incentives
14. Lack of consensus about inclusion
15. Looking like
16. Motivation of screeners
17. Need support from primary care
18. Not part of LCS
19. Opt-in opt-out model
20. Package of care
21. Perception of gen pop
22. Reduce smoking when come back for screening
23. Research evidence
24. Right thing to do
25. Screening undermines smoking cessation
26. Services for ineligible participants
27. People who smoke – resist change
28. People who smoke – want to quit
29. Some will have a history of smoking
30. Supplying patches
31. Tailored program
32. Teachable moment
33. Timing of introducing smoking cessation
34. Tobacco control
35. Training for HPs

**Participant factors (6 topics)**

**Benefits and harms (3 items)**

1. Benefits harms to participants (general)
2. Benefits
3. Harms

**Engagement and awareness (16 items)**

1. Engagement and awareness (general)
2. Advertise in different languages
3. Advertise to high-risk people
4. Co-design
5. Education needed
6. Eligibility criteria
7. External to GP
8. Funding
9. GP office, resources
10. Image of lung cancer
11. Investment in mailing, social media, sponsored adverts
12. Normalisation over time
13. Piggyback on other campaigns
14. Public transport, QR codes
15. Targeted adverts on cigarette packaging
16. Traditional advertising, TV newspapers

**Managing ineligibility (9 items)**

1. Managing ineligibility (general)
2. Ensure not diagnostic
3. Enthusiastic but not eligible
4. Maintain success of program
5. Manage people diagnosed outside program
6. People who have never smoked
7. Orders CT from GP anyway
8. Other risk factors
9. Symptom education

**Naming the Program (9 items)**

1. Naming the program (general)
2. Compare to other cancer screening programs
3. Don't use ‘cancer’
4. Health check
5. Name should reflect what it is
6. Need consumer perspective
7. Overall lung check (like LHC in UK)
8. Public understanding of screening
9. Use cancer term

**Priority populations (31 items)**

1. Priority populations (general)
2. Culturally and linguistically diverse (CALD)
3. Capture motivated
4. Characteristics
5. Complex patients
6. COVID impact
7. Decide populations
8. (Participants who) Don't go to GP
9. Empowerment, alternative pathways
10. Fear, fatalistic (beliefs and attitudes)
11. Full support for priority populations
12. Health literacy
13. Indigenous communities
14. Indigenous people have mistrust
15. Local buy-in
16. Low Socio-economic status (SES)
17. Multipronged approach to engage
18. Nihilism
19. Opportunistic (appeal to priority populations)
20. Opportunity for intervention
21. Participant to be proactive
22. Psychosocial impact
23. Relationships with primary care
24. Rural/Remote priority populations
25. People who smoke - denial
26. People who smoke - guilt
27. People who smoke - keen to know risk
28. Stigma
29. Support with follow up
30. Uptake in other screening program
31. Use existing strategies (of other screening programs)

**Recruitment and eligibility (17 items)**

1. Recruitment and eligibility (general)
2. Digital methods
3. Direct letters
4. Doorknocking rural, regional communities
5. Grassroots, population groups
6. Incentives
7. Key recruitment messages
8. “No wrong door”
9. Opportunistic
10. Patient navigators
11. Primary care
12. Quitline referral
13. Risk assessment tool
14. Self-referral into program
15. Suggestions (potential strategies for implementation)
16. Take screening to the public
17. Understanding eligibility, simpler risk assessment tool, two stage
