# Supplementary material for: “What do I think about implementing lung cancer screening? It all depends on how.” Acceptability and feasibility of lung cancer screening in Australia: The view of key stakeholders about health system factors
Source: PLoS One. 2023 Apr 5;18(4):e0283939. doi: 10.1371/journal.pone.0283939 (PMC10075440; doi:10.1371/journal.pone.0283939)
Supplement: Attachment — Submitted filename: R1 Response to reviewers PONE-D-22-30637.docx [file pone.0283939.s003.docx]

1. Please ensure that your manuscript meets PLOS ONE's style requirements, including those for file naming. The PLOS ONE style templates can be found at 
[https://journals.plos.org/plosone/s/file?id=wjVg/PLOSOne_formatting_sample_main_body.pdf](https://protect-au.mimecast.com/s/Znr6Clx1Njio8AMOYtqpUeX?domain=journals.plos.org) and 
[https://journals.plos.org/plosone/s/file?id=ba62/PLOSOne_formatting_sample_title_authors_affiliations.pdf](https://protect-au.mimecast.com/s/p34xCmO5glu53AM1QfQ4uhr?domain=journals.plos.org)

AUTHOR RESPONSE: Noted.

2. Please provide additional details regarding participant consent. In the ethics statement in the Methods and online submission information, please ensure that you have specified what type you obtained (for instance, written or verbal, and if verbal, how it was documented and witnessed). If your study included minors, state whether you obtained consent from parents or guardians. If the need for consent was waived by the ethics committee, please include this information.

AUTHOR RESPONSE: We have specified in the manuscript that consent was written.

3. In your Data Availability statement, you have not specified where the minimal data set underlying the results described in your manuscript can be found. PLOS defines a study's minimal data set as the underlying data used to reach the conclusions drawn in the manuscript and any additional data required to replicate the reported study findings in their entirety. All PLOS journals require that the minimal data set be made fully available. For more information about our data policy, please see [http://journals.plos.org/plosone/s/data-availability](https://protect-au.mimecast.com/s/-aUrCnx1jniG2KWlRHEDAze?domain=journals.plos.org).

We will update your Data Availability statement to reflect the information you provide in your cover letter.

4. Please review your reference list to ensure that it is complete and correct. If you have cited papers that have been retracted, please include the rationale for doing so in the manuscript text, or remove these references and replace them with relevant current references. Any changes to the reference list should be mentioned in the rebuttal letter that accompanies your revised manuscript. If you need to cite a retracted article, indicate the article’s retracted status in the References list and also include a citation and full reference for the retraction notice.

Additional Editor Comments:
No further comments to add; the reviewers haven't identified any major issues, instead they are largely requests for further clarification and contextualisation to help readers, particularly an international audience.

**Reviewer #1**

**Overall:** **Thank you to all authors for this interesting paper. It is of great importance to the cancer screening research. The framework provides a clear method of analysis and the findings are useful for the readers to understand how acceptable HCPs think LCS can be implemented and methods to aid the implementation.**

*AUTHOR RESPONSE:* Thank you for these positive comments.

**Abstract:**
**Pg 2 – “Focus groups were mostly held via videoconferencing” – how many were held over this method? I am guessing the others were in person could you amend and clarify?**

*AUTHOR RESPONSE:* Of the 24 focus groups, 22 were conducted via videoconference (Zoom) and two conducted in person. We have now added this to the abstract and methods.

Abstract (page 2): ‘We conducted 24 focus groups and three interviews (22 focus groups and all interviews online)...’

Data collection (page 8): ‘Twenty-two focus groups and three interviews were carried out via Zoom^TM^ and two focus groups face-to face.’

**Pg 2 – were all participants from the same region or across Australia?**

*AUTHOR RESPONSE:* Table 1 shows that participants were representative of all states and territories across Australia, with the majority located in an urban/inner-city location. We have amended the subheading in Table 1 to clarify: Australian State or Territory of Work. This has been added to the abstract.

‘We conducted 24 focus groups and three interviews (22 focus groups and all interviews online) in 2021 with 84 health professionals, researchers, and current cancer screening program managers and policy makers across all states and territories of Australia.’

**Pg 2 – “Participants took part in facilitated discussions of about one hour per group” – perhaps reword and state “Focus groups lasted approximately one hour each” to be more clear.**

*AUTHOR RESPONSE:* The authors agree with the recommendation and have made this change to the Abstract on Page 2.

**Introduction: A well written introduction, that clearly documents LC prevalence, the important of screening and the need for this research.
Pg 3 – “LCS pilot programs have been launched worldwide, including in England,”, you may discuss this later on but maybe indicate the results of the research undertaken in England on LCS**

*AUTHOR RESPONSE:* We have now added to the introduction and included some additional references (pages 4, 5):

‘The outcomes of such real-world programs show promising outcomes in terms of detection of early-stage (e.g. 81.2% Stage I or II) disease and diagnostic accuracy (e.g., 2% false positive rate; invasive surgical investigations (0.6%) in those without lung cancer) ^12^ but significant variation in uptake in high-risk populations (ranging from 4%^16^ to 52%^17^).’

**Pg 3 – missing bracket - “jurisdictions (e.g., China,12 Brazil,13 New Zealand14.”**

*AUTHOR RESPONSE:* This edit has been corrected on page 4.

**Pg 4 – the justification for using the Consolidated Framework for Implementation Research (CFIR) might this be best to have in the methods rather than in the introduction?**

*AUTHOR RESPONSE:* This has now been moved into the data analysis section on page 8.

**Methods:**
**Throughout section: Throughout the methods could you indicate which authors completed which activity using for example “Each group was moderated by a researcher with expertise in behavioural science (Initials of person).” This can be really helpful for readers to know who lead the focus groups, who moderated or did anyone make notes during the focus group, who was involved in the analysis, who conducted the interviews.**

*AUTHOR RESPONSE:* Researcher initials (RD, NR, AS) have been added to the methods on page 8.

**Throughout section:** **It is good practice to report qualitative studies in line with the Consolidated criteria for reporting qualitative research (COREQ) by Tong et al 2007 (Consolidated criteria for reporting qualitative research (COREQ): a 32-item checklist for interviews and focus groups). Could you please use and add the necessary sentence into the methods.**

*AUTHOR RESPONSE:* We have followed the COREQ checklist and included where relevant, the criteria throughout the manuscript. We have added a sentence into the methods (page 7) and included as a supplementary file:

‘This study has been reported in line with the Consolidated Criteria for Reporting Qualitative Research (COREQ; see supplementary file 1).^26^’

**Participants: No comments.
Recruitment and study processes:
Pg 5 – Consent – was this written or verbal? Was this collated prior to the date of the focus group?**

*AUTHOR RESPONSE:* Participants downloaded consent information via an online system upon expression of interest. The completed consent form was then emailed to the research team prior to the date of the focus group. This detail has been added to methods on page 7.

‘Consent involved participants downloading the consent form and emailing it to a member of the study team prior to the date of their focus group.’

**Pg 5 – “The research team members also shared study information on closed professional groups on Facebook” – how did you gain access if they were closed? Did you ask a gatekeeper or someone who was a member to circulate information?**

*AUTHOR RESPONSE:* Access was made possible due to existing memberships of these groups via the authorship team.

This detail has been added to methods on page 7:

‘Access was made possible due to existing memberships of these groups via the authorship team.’

**Focus group content:
Pg 5 – could you consider providing the semi-structured guide as a supplementary file? Or providing example of the questions/prompts within the guide.**

*AUTHOR RESPONSE:* Yes, we are happy to include this as a supplementary file.

**Data collection:
Pg 5 – “All focus groups were conducted between February and July 2021 and groups were mixed by professions.” – you have already highlighted in the recruitment that the focus groups had a mix of people (except one) maybe remove the end of this sentence.**

*AUTHOR RESPONSE:* This edit has been made on page 8.

**Pg 5 – “Groups were mostly carried out via Zoom” – guessing others were face-to-to face. Could you indicate how many were over zoom and face to face.**

*AUTHOR RESPONSE:* Of the 24 focus groups, 22 were conducted via Zoom and two conducted face-to-face. This detail has been added to page 8.

‘Twenty-two focus groups and three interviews were carried out via Zoom^TM^ and two focus groups face-to face.’

**Pg 5 – “lasted no longer than one hour” could you report the range of length and the mean.**

*AUTHOR RESPONSE:* Focus group duration ranged from 40 to 60 minutes, with a mean duration of 54 minutes. This detail has been added to page 8.

‘Focus group duration ranged from 40 to 60 minutes, with a mean duration of 54 minutes.’

**Pg 5 – “three individual interviews were conducted” – you might consider adding this into the abstract as focus groups were not the only method of data collection completed.**

*AUTHOR RESPONSE:* This has now been added to the abstract.

‘We conducted 24 focus groups and three interviews…’

**Pg 5 – “three individual interviews” – were the three interviews conducted over zoom too? – as with comment above could you indicate who completed.**

*AUTHOR RESPONSE:* This detail has been added to page 8.

‘Twenty-two focus groups and three interviews were carried out via Zoom^TM^ and two focus groups face-to face.’

**Pg 5 – “$A100”- remove the A**

*AUTHOR RESPONSE:* This edit has been made on page 8.

**Pg 5 – Did the same people lead the focus groups? I am assuming so by “moderated by a researcher” – as with comment above could you indicate who completed. Did the moderator or another member of the research team take notes/observations during the focus groups? If so, were they used in the data analysis.**

*AUTHOR RESPONSE:* Initials of researchers who led focus groups have been included on page 8.

**Data analysis:
Pg 5 – “The focus groups were recorded” – was this just audio, or video too? Were the three interviews recorded?**

*AUTHOR RESPONSE:* This was both audio and video. Details have been added to page 8.

‘The focus groups and interviews were recorded via Zoom^TM^, the audio file transcribed using TRINT^TM^ and anonymised’

**Pg 5 – “familiarised themselves with three transcripts” – were the focus group transcripts or interview or a mixture. Can you clarify in the paper?**

*AUTHOR RESPONSE:* This detail has been added to page 8.

‘independently familiarised themselves with three randomly selected focus group transcripts’

**General comment – the steps of data coding is very clear. Thank you for this!**

*AUTHOR RESPONSE:* Thank you for your positive comment.

**Results: General comments: I am not familiar with the CFIR. Therefore, I am sorry if any of the below comments are not applicable.
Table 1: the subcategories under the main heading, could you perhaps indent the categories slightly to make it easier for the reader to differentiate between the heading and sub categories.**

*AUTHOR RESPONSE:* This edit has been made to Table 1.

**Table 1: There seems to be data missing. For example, under ‘Aboriginal or Torres Strait Islander’ can you report the no and report the other countries of university education of the remaining 5 (given there are 2 sets of missing data). Perhaps have another category as you have for ‘Country of birth’?**

*AUTHOR RESPONSE:* These edits have been made to Table 1.

**Table 2: Having looked into the CFIR constructs, seems to be missing some characteristics, I can see from the references there is a 2009 reference but when I have looked there is an updated CFIR constructs. Could you possible make it clear if you are using the original framework or updated (additional file 6 -** [**https://implementationscience.biomedcentral.com/articles/10.1186/s13012-022-01245-0**](https://protect-au.mimecast.com/s/6nPbCr81nytAY1mwBCxIAt_?domain=implementationscience.biomedcentral.com)**)**

*AUTHOR RESPONSE:* We have used the original framework from 2009 and have now stated this in the introduction (page 6):

‘The objectives were to gain an understanding of the perceptions and attitudes of health professionals about LCS implementation and to identify and analyse the potential barriers and facilitators using the original Consolidated Framework for Implementation Research (CFIR).^25^’

**Table 3: Thank you for providing a quote table. This is really helpful. I particularly like that you have provided more than one quote from different focus groups and participants. Could you perhaps do this for the domains where only one is present?**

*AUTHOR RESPONSE:* Where another quote was available to represent a topic, we have now added this in.

**Domains: While I like the table with the quotes in, I think integrating the quotes into the narrative itself would be beneficial to support your interpretations and can also help the reader understand some of the narrative for example “Participants raised some questions about whether there was evidence behind the cost effectiveness of LCS.” – what were the questions raised? I appreciate that word count will be difficult and the amount of the domains and sub-categories that are being reported.**

*AUTHOR RESPONSE:* We have now integrated the quotes into the main body of the manuscript and removed Table 3.

**General comment: Your results are really interesting.**

*AUTHOR RESPONSE:* Thank you.

**Discussion:
Pg 23 “one of the most comprehensive internationally studies” – international?**

*AUTHOR RESPONSE:* This edit has been corrected on page 31.

**Pg 25 “Breast Screen does not” – breast cancer screening?**

*AUTHOR RESPONSE:* BreastScreen refers to the name of the national breast cancer screening program, clarified on page 34.

**Pg 27 – thank you for acknowledging the limitation that this paper does not involve LCS participants, but signposting the reader to your paper. This is really helpful and provides a balance on the implementation pf LCS in Australia.**

*AUTHOR RESPONSE*: Thank you for your positive comment.

**Figure 1: Can you highlight what the orange bubbles mean in the figure? You have highlighted what the dotted line is but the a key of the colours would be useful.**

*AUTHOR RESPONSE:* The dark blue circles are topics and the orange are cross cutting topics across factors. This has now been added to the figure.

**Reviewer #2: Overview**

**A topical paper with some interesting findings, which will be key in understanding whether there is a place for lung cancer screening in Australia, as well as more generally worldwide. Of particular interest are repeating participant concerns relating to test specificity and patient pathways, e.g. recall rates, incidental findings, funding, access, and the impact of referral and treatment on current systems.

I am not familiar with the healthcare provision in Australia and would have liked some brief scene setting as to the structure of the different funding and commissioning streams.**

*AUTHOR RESPONSE:* We have included a sentence about the healthcare system model in Australia on page 5:

‘The Australian healthcare system is a hybrid model. Citizens, permanent residents and refugees are given public insurance known as Medicare, but can also buy private insurance coverage and gain access to privately operated health facilities.^20^ While the Australian Government determines whether new screening programs are implemented, state and territory governments are typically responsible for the planning and performance of screening program and healthcare facilities.^21^ This results in complex policy decision making about how best to resource new screening programs.’

**I like the idea of a quote in the title of the paper, but I’m not sure I understand the one that’s been chosen.**

*AUTHOR RESPONSE: As the reviewer struggled to understand the quote, we have substituted it with another choice. The title of the manuscript is now:* “What do I think about implementing lung cancer screening? It all depends on how.” Acceptability and feasibility of lung cancer screening in Australia: the view of key stakeholders about health system factors.

**I think it is important to include in your introduction some data from diagnostic accuracy studies of Computed Tomography (CT) and Low Dose Computed Tomography (LDCT), as well as a basic explanation of the two different tests. Include information about radiation risk, and why it might be important for a test to be ‘low dose’ in a screening population, discuss risk versus benefit and why high-risk populations only are currently being screened. Also, include any health economics and cost-effectiveness data published (if there is any) about the cost effectiveness of Lung Cancer Screening (LCS) programmes.**

*AUTHOR RESPONSE:* We appreciate this suggestion and have made some modifications to include mention of low-dose CT being the clinically effective test use in high-risk populations in the first paragraph. We have not provided a comprehensive background analysis about the test, benefits and harms and cost effectiveness in the introduction as this is well documented elsewhere in the literature.

Introduction

A nice overview is provided of the worldwide status of LCS programmes in various countries, but I would be interested to see more detail around this, such as are they using CT or LDCT, how often, in which populations, is there published data regarding outcomes etc.

*AUTHOR RESPONSE:* Thank you for this suggestion. As this is a rapidly changing field and LDCT is the standard test used internationally, we have made reference to the latest reports and reviews, as this task is out of scope with the purpose of the manuscript.

**The third paragraph mentions a recommendation by ‘the Medical Services Advisory Committee’, some context might help explain who they are and why they made this recommendation.**

*AUTHOR RESPONSE:* We have added an explanation as to who they are and why they made this recommendation into the introduction (pages 4, 5):

‘In October 2022, the Medical Services Advisory Committee (MSAC; an independent non-statutory committee of the Australian Government Department of Health and Aged Care that appraises new medical services proposed for public funding, and provides advice to Government on whether a new medical service should be publicly funded) recommended funding a national program after considering the strength of the available evidence in relation to comparative safety, clinical effectiveness, cost-effectiveness and total cost, with Australian Government endorsement yet to be finalised.^19’^

**Some interesting findings from other international studies are mentioned in the next paragraph, with barriers such as ‘fear and stigma’, can you explain these a little more. Fear and stigma of what? ‘Scepticism about evidence of benefits’, who is sceptical? Are these worries justified? Diagnostic accuracy figures could be used to illustrate how effective an LCS programme can be in the correct population. At the end of this paragraph the sentence beginning, ‘Further evidence is needed to understand whether LCS …’ is a long and complex sentence that is difficult to read and understand, the authors could consider splitting it up to make it clearer.**

*AUTHOR RESPONSE:* These have now been clarified and the final sentence broken into two. We have added in data about diagnostic accuracy into the introduction (page 4):

The outcomes of such real-world programs show promising outcomes in terms of detection of early-stage (e.g. 81.2% Stage I or II) disease and diagnostic accuracy (e.g., 2% false positive rate; invasive surgical investigations (0.6%) in those without lung cancer) ^12^ but significant variation in uptake in high-risk populations (ranging from 4%^16^ to 52%^17^).

**Paragraph four, which outlines the aims and objectives and introduces the ‘Consolidated Framework for Implementation Research’, feels a little confused. Consider moving the explanation of the framework to the methods data analysis section, sentence beginning, ‘This conceptual framework has been developed to…’.**

*AUTHOR RESPONSE:* This has now been moved into the data analysis section on page 8.

**Methods

The results section felt a little repetitive at times, perhaps due to the mapping of results into the CFIR framework, justification for the use of this framework in this section would help.**

*AUTHOR RESPONSE:* We have moved the justification for use of the CFIR from the introduction into the methods and have moved the quotes into the manuscript.

**In ‘participants’, the authors state ‘there were no specific exclusion criteria’, explain why not and how participants were selected instead. Edit: a ‘passive snowballing approach’ is mentioned later, it might be better to include these two points together.**

*AUTHOR RESPONSE:* There was no specific exclusion criteria as we wanted to include a range of disciplines from across Australia. The suggested edit has been made on page 6.

‘There were no specific exclusion criteria as we wanted to include professionals from multiple disciplines and across Australia, and a passive snowballing approach was used, whereby focus group participants could recommend other colleagues to invite. No prior knowledge about LCS was required of participants.’

**In ‘recruitment and study processes’ the authors state participants were recruited from ‘regional, remote and urban settings where lung cancer incidence is higher than in metropolitan settings’. Can simpler language be used here to make this easier to understand, for example, use the same language as in Table 1, ‘urban/inner-city, suburban and rural’?**

*AUTHOR RESPONSE:* We have amended the text on page 6 to read ‘regional, rural and remote settings’, as ‘regional, rural and urban’ was incorrect. We would prefer to keep the description in Table 1 as it is, as these were the options participants were given in the demographic questionnaire.

**The rest of this paragraph explains the excellent design of the study and the writing is clear and easy to understand.**

*AUTHOR RESPONSE:* Thank you for this positive comment.

**The ‘Focus group content’ paragraph states that a presentation was used which was based on the findings from some international LCS randomised controlled trials, an Australian LCS enquiry and examples of international LCS programmes – could some if this data be included in the introduction? I find myself wondering what these findings are.**

*AUTHOR RESPONSE:* The findings of the international RCTs have been presented in the introduction previously, and the findings of the enquiry have now been added (page 4):

‘This enquiry estimated that 580,000 Australians would be eligible for screening on completion of a risk assessment tool, over 12,000 deaths would be prevented and over 70% of all screen detected lung cancer would be diagnosed at an early stage.’

**Were any of the topic guides reflected upon and then altered between groups to improve?**

*AUTHOR RESPONSE:* The structured presentation was modified throughout the groups to streamline the groups and ensure all content was covered. This has now been explained in the manuscript (page 7):

‘This presentation was adapted between group events as it became evident which information was most important to cover in the groups and to reduce the time burden on participants.’

**‘Data collection’ includes a sentence, which states ‘for those who could not attend a focus group, three individual interviews were conducted.’ This is slightly misleading, do they mean three interviews per participant or three participants each having one interview? There is only one sentence about the researcher conducting the groups/interviews. I’d like to know more about their background/position and their potential effect on participants and the data. By extension, are the researchers who undertook the analysis the same as those undertaking the data collection? I’d like to see more detail on the specific researchers and their roles within the data collection and analysis. Edit: I found this at the end of the paper, but it might be nice to explain briefly here?**

*AUTHOR RESPONSE:* This has now been clarified on page 8:

‘Three participants were not able to attend a focus group and so they each participated in individual interviews conducted via Zoom^TM^.’

We have also added further details about the research team and added the initials throughout the methods of the authors conducting the focus groups, interviews and data analysis.

**Results

Table 1 – why are there so many participants in ‘other’ categories, such as 14 professional roles and 16 workplace settings?**

*AUTHOR RESPONSE:* Participants’ roles and workplaces covered the entire spectrum of expertise from clinical to screening program staff, we have represented here the most frequent responses.

**Although Table 3 is a nice summary, the quotes feel ‘disembodied’ and I would like them to be embedded within the relevant text. It was difficult to go back and forwards between the table and the text when prompted to find illustrative quotes. Some words are in quotation marks within the body of text, but I was unsure if these are quotes from the data because they didn’t include participant IDs.**

*AUTHOR RESPONSE:* We have now moved the quotes into the main body of the manuscript and out of table 3. Any quote given in the manuscript is accompanied by the focus group ID and the profession listed, rather than individual IDs.

**I am not sure how Table 2 fits in or what it adds. Could information from the table be explained within the body of the text alongside the relevant quotes to give more context and a better flow?**

*AUTHOR RESPONSE:* Table 2 has been included to provide details about each construct of the CFIR, especially to readers who will not be familiar with the CFIR. Table 2 also details which topics have been mapped to each construct, alongside the short description so that readers can refer across. We originally had it alongside the quotes but the table became very large and so decided to split this.

**Table 3:
• CFIR construct ‘complexity’, Quote 1 – please explain the Australian healthcare system somewhere and what the state and federal government divide is.**

*AUTHOR RESPONSE:* The Australian healthcare model has now been explained in the introduction (page 5).

‘The Australian healthcare system is a hybrid model. Citizens, permanent residents and refugees are given public insurance known as Medicare, but can also buy private insurance coverage and gain access to privately operated health facilities.^20^ While the Australian Government determines whether new screening programs are implemented, state and territory governments are typically responsible for the planning and performance of screening program and healthcare facilities.^21^ This results in complex policy decision making about how best to resource new screening programs.’

**• CFIR construct ‘cosmopolitan’, - I don’t understand this quote.**

*AUTHOR RESPONSE:* The quote provided reflects ‘The degree to which an organization is networked with other external organizations.’ This quote demonstrates that hospitals are networked with radiology services and that LCS doesn’t need to be done just in a hospital but can also be done within radiology services.

**• CFIR construct ‘external policy and incentives’, Quote 2 – discuss somewhere the ethical implications of offering incentives to take part in a screening programme which has potential to cause harm and the impact of incentives on fully-informed shared-decisions in healthcare.**

*AUTHOR RESPONSE:* We have acknowledged this in the discussion, as there is a greater need for understanding incentives with lung cancer screening (page 32):

‘The potential persuasive nature of financial incentives directed at participants or providers need to be considered alongside their impact on participants making a fully informed shared decision with their health professional about taking part in LCS.’

**• CFIR construct ‘readiness for implementation’, both quotes in ‘workforce and programme delivery’ and Quote 2 from ‘primary care education’ relate indirectly to the specificity of CT/LDCT (and resulting recall rate and biopsy rate). As I’ve said, I think it is important to include some review of these figures from the literature in your introduction and then you can discuss this in the context of these quotes.**

*AUTHOR RESPONSE:* We have now included information about diagnostic accuracy in real-world programs in the introduction and made a further reference to this in the discussion on page 33.

**Results section ‘cost’, the sentence ‘costs in the set-up of the programme …’ doesn’t make sense to me.**

*AUTHOR RESPONSE:* This has been amended to say ‘costs to set-up the program’.

**In ‘peer pressure’, NLST criteria is mentioned, what is this?**

*AUTHOR RESPONSE:* NLST stands for National Lung Screen Trial and has been clarified on Pages 22,23.

**In ‘workforce and program delivery’, ‘telehealth’ is mentioned, what is this?**

*AUTHOR RESPONSE:* Telehealth is having a consultation with a healthcare provider by phone or video call and has been clarified on page 29:

‘Telehealth, a consultation with a healthcare provider by phone or video call, was offered as a strategy for managing patients across the screening pathway.’

**In ‘primary care engagement and education’, the opening sentence ‘Participants viewed GPs as the gatekeepers to LCS.’ I’m not sure I understand what is meant by this and the implications.**

*AUTHOR RESPONSE:* ‘Gatekeeper’ in this context means a person or thing that controls access to something and has been clarified on page 26:

‘Participants viewed GPs as the person that controlled patients’ access to LCS.’

**In ‘reflecting and evaluating’, AI is mentioned for the first time, it would be nice to expand upon this in the discussion in light of the rise of AI used to ease workload pressure in other areas of cancer screening. There is a brilliant quote I read elsewhere in the manuscript, which could be used here about ‘boring CT reading’.**

*AUTHOR RESPONSE:* We have expanded upon the use of AI on page 33:

‘The potential use of AI was discussed to aid the reading of scans while easing the workload pressure and being able to automate tedious tasks. Research in the area of AI and cancer screening is rapidly evolving, with a study demonstrating improved sensitivity and specificity when using of a ‘decision-referral’ approach to reading mammograms, combining the strengths of AI and radiologists.^35^ AI has also been shown to perform favourably when predicting risk of malignancy in pulmonary nodules.^36^ If to be used in a potential LCS program, future research will be required to evaluate which approach will be most effective and meet quality assurance standards.’

**Discussion

In paragraph four, the authors mention a pilot trial, the ‘International Lung Screening Trial’ running in Australia (among other countries), so it’s already happening?**

*AUTHOR RESPONSE:* The International Lung Screening Trial aimed to 1) compare the predictive accuracies of the PLCOm2012 and the USPSTF screening selection criteria and 2) evaluate a nodule management protocol based on the use of the Pan Can nodule probability calculator on baseline screening LDCT. The aim of this trial was not to test implementation of lung cancer screening and has been in a limited sample of 4500 participants. We have referenced the protocol paper in the manuscript (Lim et al, 2020 doi:10.1513/AnnalsATS.201902-102OC).

**Paragraph five suggests the need for ‘patient and professional champions’, which is a lovely phrase. The sentence about a ‘bottom up approach’ being more effective than a ‘top down approach’ could be clearer.**

*AUTHOR RESPONSE:* We have amended and clarified this sentence (pages 34,35):

‘Local champions have been key facilitators to LCS implementation in the US,^34,42^ with findings that a ‘bottom up’ approach, gaining buy in and input from participants and frontline staff responsible for implementation, was more effective than a ‘top down’ approach.^29^’

**In strengths and limitations, the authors mention that participants more knowledgeable about LCS may have been recruited than not, why is this and what is the effect, if any, on the data?**

*AUTHOR RESPONSE:* We have expanded on this in the limitations (page 35):

‘However, we may have attracted more participants who were knowledgeable about LCS than not due to the nature of recruitment for studies around a particular topic, and we did not formally measure their knowledge prior to focus groups taking place. Therefore, those participants with more knowledge may have felt more comfortable speaking to the feasibility and acceptability than those who did not. However, valuable and new perspectives were gained from those who had no knowledge of LCS prior to the study.’
