# Supplementary material for: “What do I think about implementing lung cancer screening? It all depends on how.” Acceptability and feasibility of lung cancer screening in Australia: The view of key stakeholders about health system factors
Source: PLoS One. 2023 Apr 5;18(4):e0283939. doi: 10.1371/journal.pone.0283939 (PMC10075440; doi:10.1371/journal.pone.0283939)
Supplement: Attachment — Submitted filename: Response to reviewers PONE-D-22-30637 R2.docx [file pone.0283939.s004.docx]

| Postal address:  207 Bouverie Street, Carlton, VIC, 3010, Australia | Email:  nicole.rankin@unimelb.edu.au |
| --- | --- |

**Response to reviewers – R2**

**4. Have the authors made all data underlying the findings in their manuscript fully available?**

**Reviewer #1: No**

**Reviewer #2:** Yes

**Response:** The authors wish to advise that an updated response and cover letter was sent through on 24 Feb in response to their policies about data sharing and funding acknowledgements.

**6. Review Comments to the Author**

**Reviewer #1:** Overall: I want to thank the authors for the significant changes that have been completed in this revision. They have greatly improved the manuscript. There are a few minor additional comments below, other than those I have no further comments.

Pg 3 – “and over 70% of all screen detected lung cancers would be diagnosed at an early stage” – Sorry I am not sure this makes sense upon review.

**Response:** This has been amended to read, ‘of all screen-detected lung cancer, over 70% would be diagnosed at an early stage’ (Pg 3).

Pg 4 - “barriers to lung cancer screening at participant” – change to LCS in line with the aforementioned abbreviation

**Response:** This has been amended to ‘LCS’ (Pg 4) as suggested.

Pg 6 – “Data collection: All focus groups and interviews were conducted between February and July 2021..” – double full stop

**Response:** This has been corrected (Pg 6) as suggested.

Pg 9 – “Other 7 (8.5))” – double brackets

**Response:** This has been corrected (Pg 9) as suggested.

Pg 23 – “(FG2, Thoracic Surgeon” – missing bracket

**Response:** This has been corrected (Pg 23) as suggested.

Pg 24 – “A need to learn what hasn’t” – ‘has not’ instead of ‘hasn’t’ (sorry this was not identified initially)

**Response:** This has been corrected (Pg 24) as suggested.

Pg 26 – “(FG26, Chief Executive” – missing bracket

**Response:** This has been corrected (Pg 26) as suggested.
